# New method for visualizing the dose rate distribution around the Fukushima Daiichi Nuclear Power Plant using artificial neural networks

**DOI:** 10.1038/s41598-021-81546-4

**Published:** 2021-01-20

**Authors:** Miyuki Sasaki, Yukihisa Sanada, Estiner W. Katengeza, Akio Yamamoto

**Affiliations:** 1 Collaborative Laboratories for Advanced Decommissioning Science, Japan Atomic Energy Agency, 45−169 Sukakeba, Minamisoma, Fukushima 975-0036 Japan; 2 Department of Environment Systems, The University of Tokyo, 7-3-1 Hongo, Bunkyo-ku, Tokyo, 113-8654 Japan; 3 Graduate School of Engineering, Department of Applied Energy, Nagoya University, C2-4 Furoucho, Chikusaku, Nagoya, 464-8603 Japan

**Keywords:** Environmental monitoring, Mathematics and computing

## Abstract

This study proposes a new method of visualizing the ambient dose rate distribution using artificial neural networks (ANNs) from airborne radiation monitoring results. The method was applied to the results of the airborne radiation monitoring which was conducted around the Fukushima Daiichi Nuclear Power Plant by an unmanned aerial vehicle. Much of the survey data obtained in the past were used as the training data for building a network. The number of training cases was related to the error between the ground and converted values by the ANN. The quantitative evaluation index (the root-mean-square error) between the ANN-converted value and the ground-based survey result converged at 200 training cases. This number of training case was considered a rough criterion of the required number of training cases. The reliability of the ANN method was evaluated by comparison with the ground-based survey data. The dose rate map created by the ANNs method reproduced ground-based survey results better than traditional methods.

## Introduction

Large quantities of radionuclides were released into the atmosphere after the Fukushima Daiichi Nuclear Power Plant (FDNPP) accident in March 2011^1^. Nine years after the accident, the ambient dose rate (air dose rate) has been decreased by radioactive decay, decontamination work, and weathering effect within the 80-km radius zones from the FDNPP^2^. As a quick and efficient survey method, manned helicopters and unmanned aerial vehicles (UAVs) have been developed to visualize the environmental distribution of the air dose rate in the airborne radiation survey (UAV-survey) after the FDNPP accident^3,4^. UAVs are effective tools for data collection over wide areas that are located around FDNPP because a person does not have to approach a dangerous place^5,6^. However, a UAV-survey is less accurate than a ground-based radiation survey using a handheld survey meter because it has a long distance from the ground surface source.

In a conventional approach, the count rates and the pulse height distributions collected by the UAV-survey are converted into air dose rates and deposition of radioactive cesium on the ground. In this approach, the ground is assumed to have a flat form, and this is called the flat source model (FSM)^7^. Improving UAV-survey accuracy requires accounting for the topographic features in radiation survey areas. Some reports suggested that the topographic effect was corrected by published big topographical data, such as the digital elevation model (DEM) and the digital surface model (DSM)^8,9^. A conversion method using the maximum-likelihood expectation-maximization (ML-EM) method was recently proposed to convert the value measured from the sky into an air dose rate of 1 m above the ground level (agl.)^10,11^. The conversion using the ML-EM method requires the creation of many types of attenuation parameters to improve the conversion accuracy. This method is effective for conversion in complex terrain areas, such as mountains or forests, which could not be reproduced by conventional FSM methods. However, the decision on the parameters needs many on-site measurement experiments or simulation work.

**Figure 1:**
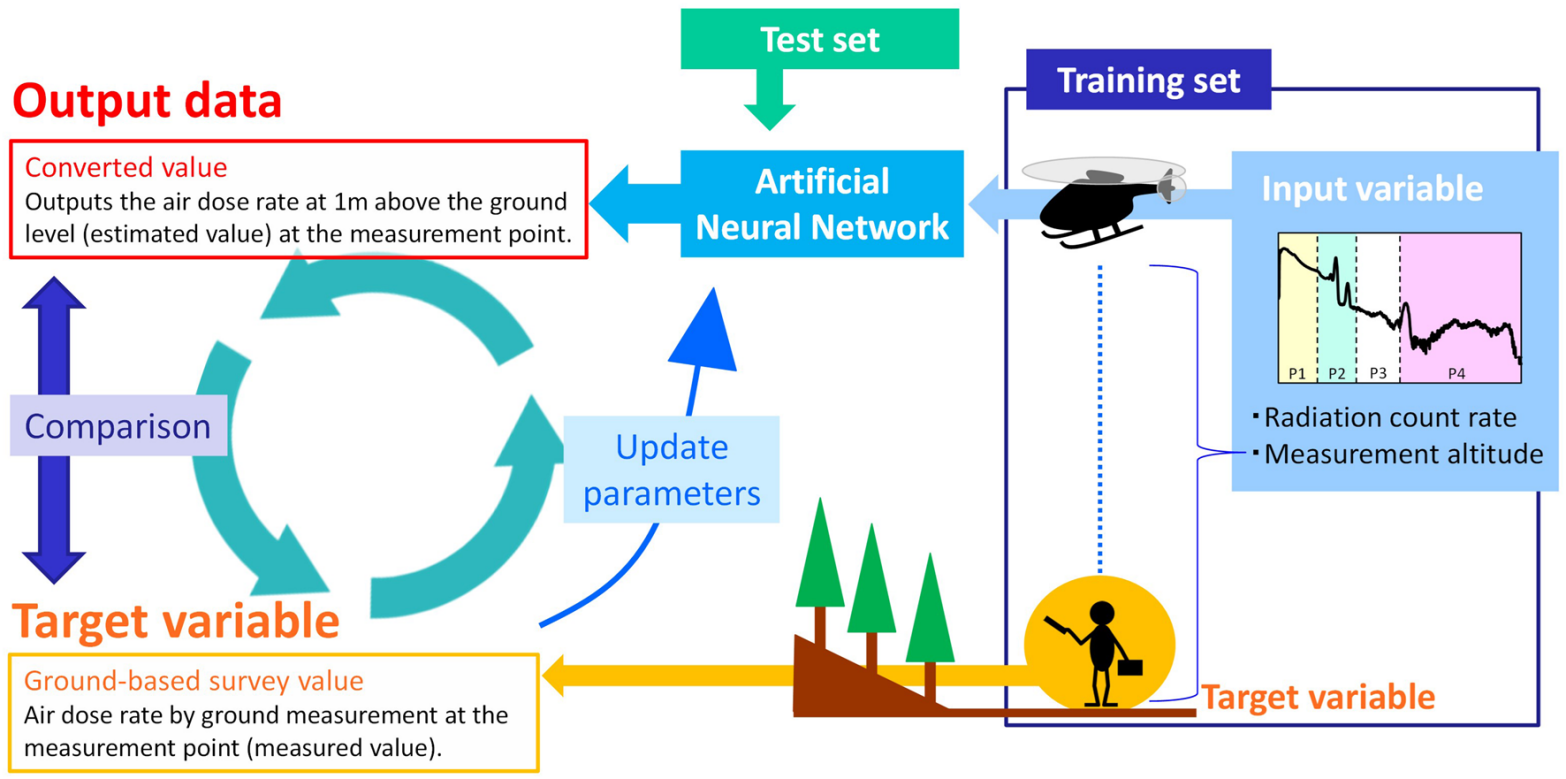
Images of the application of the artificial neural network to the airborne radiation survey.

To solve this problem, we attempted herein to convert radiation measurement values using an artificial neural network (ANN). The ANN is applied to the study fields of medicine, physics, etc. as a kind of deep learning^12,13^. Simple and easy ANN software was recently published^14,15^. Moreover, the ANN method was applied to search for radiation sources for nuclear security^16^. Compared to the ML-EM method, the conversion by an ANN is expected to reduce both the workload of creating many parameters and the calculation time (Fig. 1). We have many data sets from the UAV-survey and the ground-based survey in the same locations because periodical surveys were conducted around the FDNPP as part of the national project^4–6^. These data sets were suitable for the training set in the ANN with published topographical data^17^. The air dose rate maps created by the UAV-survey are used for emergency monitoring data and determine the evacuation route of inhabitants; thus, the reliability of the created air dose rate map must be evaluated.

We developed herein a new conversion method using the ANN to improve the accuracy and the analysis time of the UAV-survey. This ANN method was optimized by evaluating the number of hidden layers constituting a network. In addition, we also evaluated the relationship between the number of data cases used for the training and the error between the ANN-converted UAV-survey and ground-based survey values. Finally, the ANN method validation was evaluated by comparison with the FSM and the ML-EM method.

## Materials and methods

### ANN method

**Figure 2:**
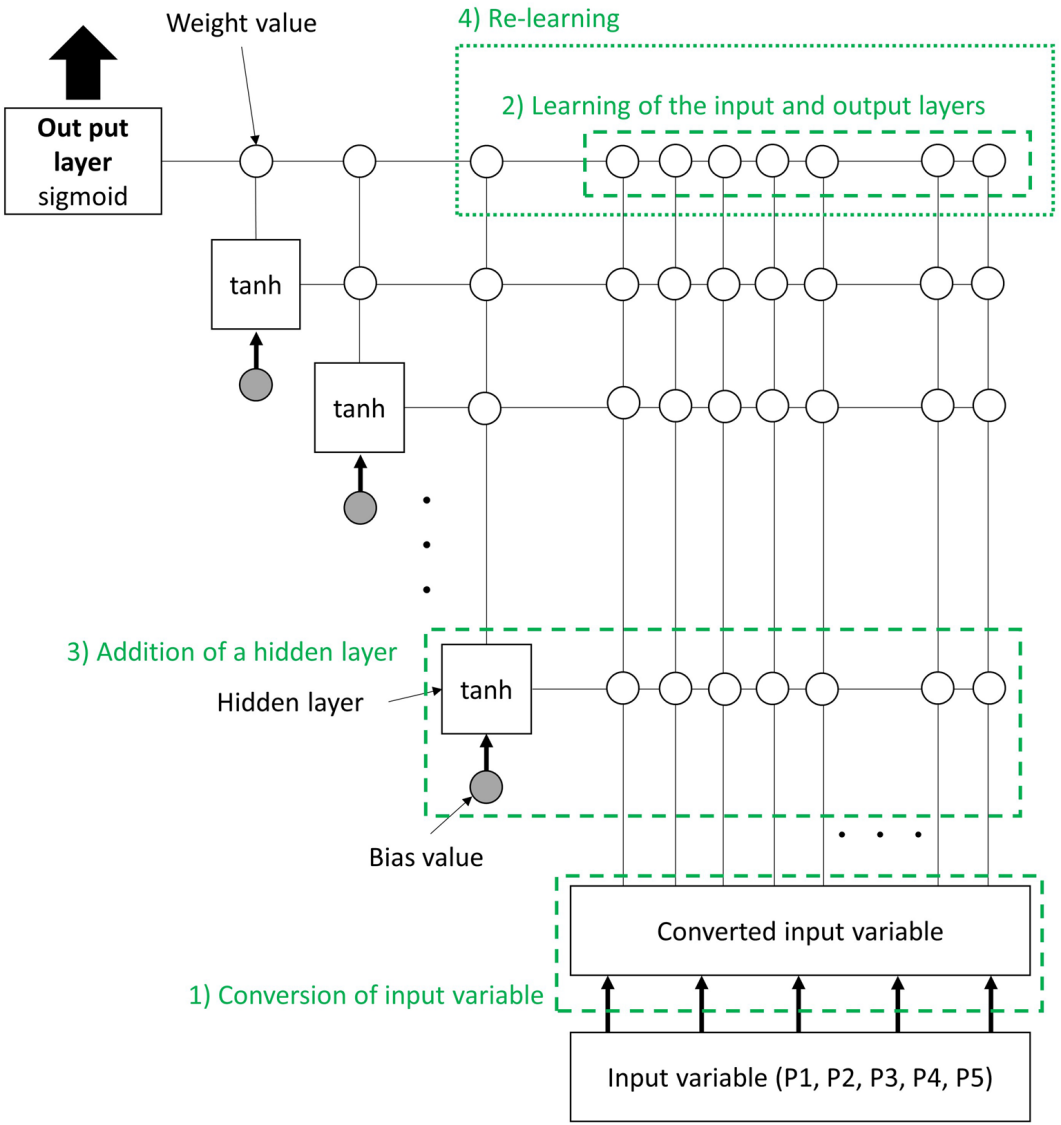
Flow of the network construction. The network was constructed by cascade correlation. The weight update method used was the adaptive subgradient method. The objective function was cross-entropy with added ridge regression.

In this study, the ANN was constructed using NeuralWorks Predict (NeuralWare, Carnegie, USA), a software that uses cascade correlation to determine the optimal network structure with a simple graphical user interface^18^. This learning is classified as a regression problem of supervised learning. The cascade correlation is a constructive learning rule and a supervised learning algorithm that constructs a feed-forward network. Learning starts with a minimal network consisting only of the input and output layers. Minimizing the overall error of a network, new hidden layers are then added step by step. The cascade correlation builds a network in which one hidden layer has one neuron. Figure 2 depicts the ANN modeling flowchart. One training datum (set of input and target variables) is defined as a “training case.” A dataset of multiple training cases is defined as a “training set”.

The training set was randomly divided into two cases depending on the purpose of this method. In the ANN construction, 70% of all the training cases were randomly selected. The other training case was used to evaluate the calculation of the correlation coefficient with the target variable. The changes in the evaluation were observed each time the learning was repeated. Learning was stopped when the correlation coefficient stopped improving. The construction flow of the network is presented below and in Fig. 2:

1. Conversion of the input variable: The input variable is represented by some functions. The data with a high correlation between the input and target variables are normalized and added as the input data.
2. Learning of the input and output layers: Training is first implemented only on the input and output layers without the hidden layer. The output layer is a sigmoid function recommended from NeuralWorks Predict.
3. Addition of a hidden layer: If the index of the reliability (the correlation coefficient) of the output value in the training of former procedure 2) was not enough, one hidden layer would be added automatically. All hidden layers are tanh functions recommended from NeuralWorks Predict.
4. Re-learning: Learning will be performed again with the added hidden layer. The connection is fixed between the hidden and input layers, and the weight values are not updated. One hidden layer will be added again if the output vale is not improved. When learning is judged to be sufficient, the hidden layer addition is stopped, as well as learning.

The neural network was constructed with simple input variables and one hidden layer function for judging whether the UAV-survey data can be converted by the ANN. The output value of the network was compared with the target variable using the correlation coefficient (*r*) which is defined as a criterion for sufficient learning. The learning was continued until no improvement of *r* was observed. *r* can be expressed as follows:

1 $$r = \frac{{\mathop \sum \nolimits_{i}^{n} \left( {x_{i} - \overline{x}} \right)\left( {y_{i} - \overline{y}} \right)}}{{\sqrt {\left( {\mathop \sum \nolimits_{i}^{n} \left( {x_{i} - \overline{x}} \right)^{2} } \right)\left( {\mathop \sum \nolimits_{i}^{n} \left( {y_{i} - \overline{y}} \right)^{2} } \right)} }}$$

where *n* is the total number of data; *x* is the converted value; *y* is the ground-based survey value (normalized target variable); and $\overline{x}$ and $\overline{y}$ are the averages for each value. The weight update method used was the adaptive subgradient method^19^. The objective function was cross-entropy with added ridge regression.

### Training set

**Figure 3:**
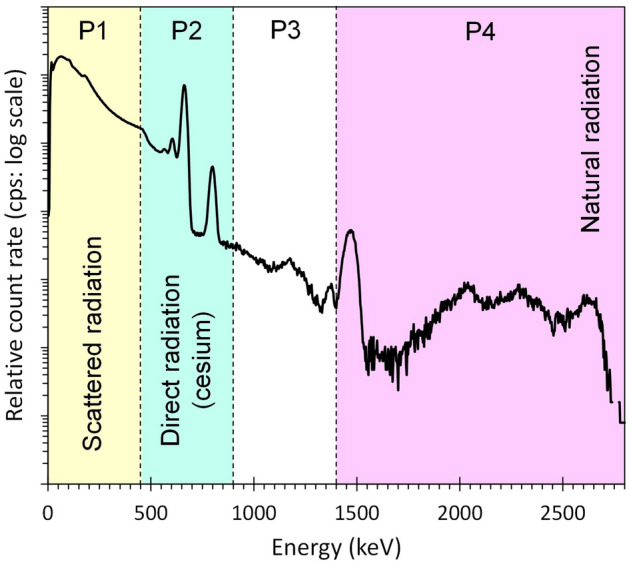
Typical gamma-ray spectrum of a UAV spectrometer with a LaBr3 (Ce) sensor. This spectrum was obtained at 50 m agl. around the FDNPP.

The radiation measurement data used for the ANN were acquired by the UAV (i.e., in this case, a type of unmanned helicopter from FAZER-R-G2, Yamaha Motor Co. Ltd., Shizuoka, Japan) around the FDNPP at the fiscal year of 2018 and 2019. The UAV was originally developed for spraying pesticides but was later adopted for radiation measurements. This UAV is operated manually during takeoff and landing and has a program operator for autonomous flight and an operator for the radiation detector. The UAV can conduct a programmed flight with the help of detailed self-localization using a real-time kinematic global positioning system. Its flight waypoints and altitude can be set. The detailed specifications of the UAV are given in a past research^4,5^. The dedicated radiation detector consisted of a LaBr_3_ (Ce) scintillation detector (38 mm $\upphi$  × 38 mm H × three detectors) and detected gamma-rays in the energy range of 50–2800 keV (Japan Radiation Engineering Co. Ltd., Ibaraki, Japan). Figure 3 depicts a typical gamma-ray spectrum. The UAV typically uses 50 m, 5 m s^−1^ and 50 m agl. as the flight line space, flight speed and flight altitude, respectively. The typical flight altitude used to obtain the training set was 80 m, but the actual altitude above the ground ranged from approximately 40 m to 240 m to avoid buildings, mountains, steel towers, etc. A photographic survey using an unmanned helicopter was also conducted to construct a 3D orthophoto map for calculating the flight altitude above the ground or object surface. Each datum was averaged over a 10 m mesh.

**Figure 4:**
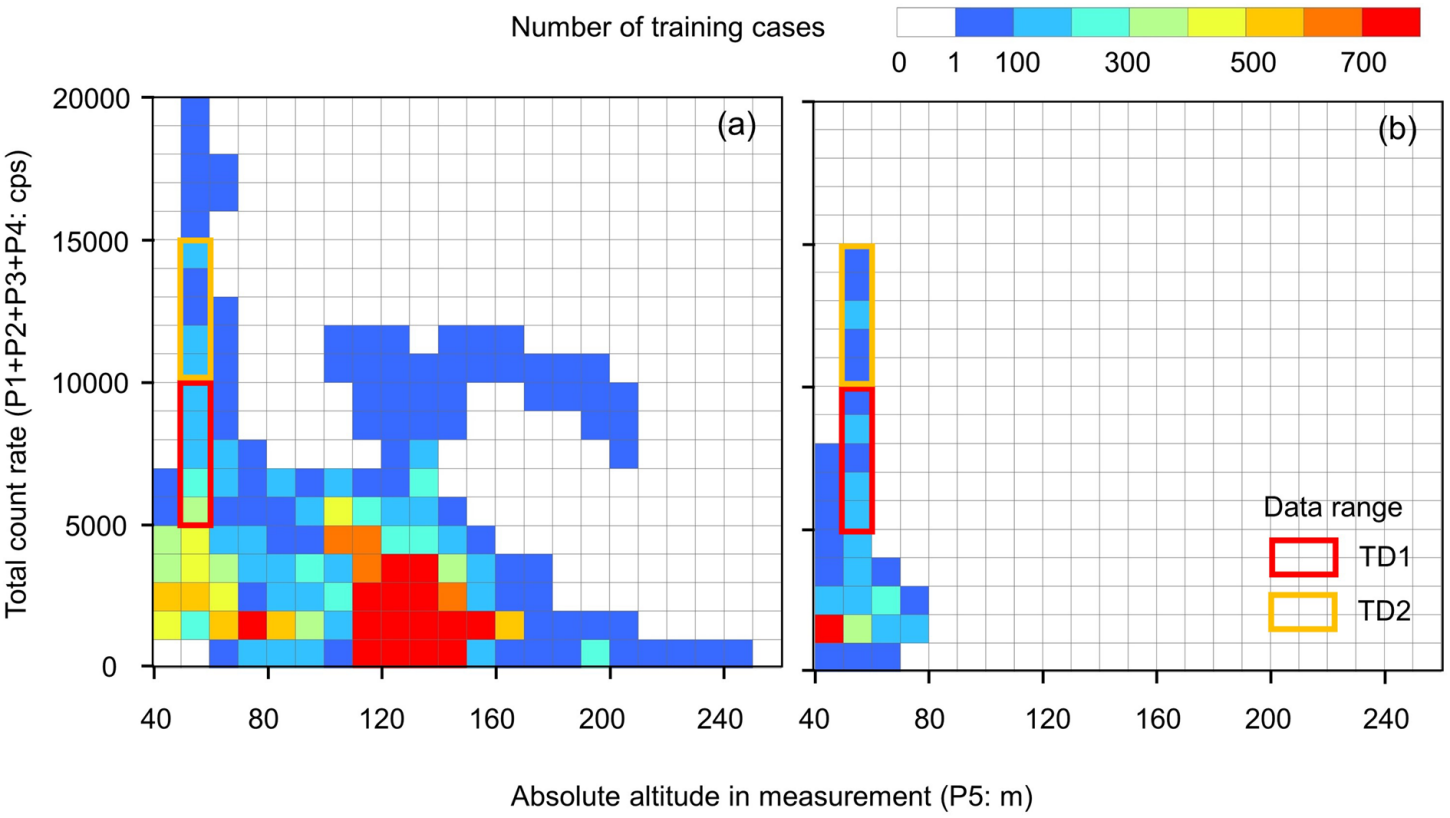
Characteristics of the (**a**) training set (37,936 cases) and the (**b**) test set (3442 cases). The areas of TD1 and TD2 are the training cases presented in Fig. 6.

Table 1 summarizes the input variables (training set). The count rate (i.e., P1, P2, P3, and P4) was obtained by dividing the gamma-ray spectrum into four regions (Fig. 3) to separate the following components: scattered radiation, P1; direct radiation from radiocesium, P2; and natural radiation, P3 and P4. P5 is the absolute altitude of the UAV, which is a major factor used to assess the air attenuation of gamma radiation. The absolute altitude was calculated by subtracting the surface altitude (DEM) from the flight altitude above sea level using the global navigation satellite system. The air dose rate at 1 m agl. (T1) was used as the target variable for the ANN and acquired using a handheld CsI (Tl) (38 mm L × 38 mm W × 25 mm H) scintillation detector (Hot spot finder, Japan Shield Technical Research Co. Ltd., Fukushima, Japan). The training set (input and target variables) had 37,936 cases (Table 1 and Fig. 4a). Figure 4a illustrates a three-dimension scatter diagram of the total count (P1 + P2 + P3 + P4), which is as an index of the radiation intensity, and the absolute altitude (P5) to enable us to understand the training set distribution. In this figure, the number of training cases on 120–150 m agl. of the absolute altitude in the measurement was relatively higher. In contrast, the condition of both high radiation intensity and high absolute altitude in the measurement was relatively small.

**Table 1:** List of training and test sets.

Data type	Survey method	Parameter	Unit	Data range (Min–Max)		Description
				Training set (37,936 sets)	Test set (3442 sets)	
Input variable	**UAV-based survey data**					
		P1	cps	301–17,396	640–13,069	Count rate of 50–450 keV Scattered radiation
		P2	cps	35–1663	70–1350	Count rate of 450–900 keV Direct radiation from radiocesium
		P3	cps	14–27	16–26	Count rate of 900–1400 keV U and Th series natural radiation
		P4	cps	13–26	18–26	Count rate of 1400–2800 keV ^40^K, U and Th series natural radiation
	**GPS position data**					
		P5	m (agl.)	44–244	43–75	Absolute altitude in measurement (Flight altitude)—(Surface altitude)
	**Ground-based survey data**					
Target variable		T1	$\upmu$Sv h^-1^	0.064–13	–	For training
Validation data		V1	$\upmu$Sv h^-1^	–	0.12–10	For validation

### Test set

The test set different from the training set was used to evaluate the constructed ANN. The test set was obtained from Futaba-machi, which is located 3 km northwest from the FDNPP. This study area (1 km × 2 km) is still acknowledged as an evacuation area by the Japanese government. The flight line space, flight speed, and flight altitude of the UAV used were 50 m, 5 m s^−1^, and 50 m agl., respectively. For the output value validation, the ground-based survey data (V1) were obtained using a handheld survey meter at the same time as the UAV flight. All data were obtained for less than 8 h on August 19, 2018. Each datum was averaged over a 10 m mesh.

The test set had 3442 cases (Table 1). For comparison with the distribution of the training set, a three-dimensional scatter diagram of the total count (P1 + P2 + P3 + P4) and the absolute altitude (P5) was shown in Fig. 4b. The average flight altitude of the test set was 50 m. However, the actual altitude above the ground ranged from approximately 40 m to 80 m due to the avoidance of obstructions. Almost all test cases were understood to be within the range of the training set (Fig. 4a,b).

### Evaluation of the neural network condition

The accuracy of the ANN method was expected to depend on the neural network condition (e.g., number of hidden layers and a distribution range of the training set). We conducted some verification tests to determine the effectiveness of the output validation by the neural network condition.

First, the output value validation was evaluated by limiting the number of hidden layers. NeuralWorks Predict automatically creates a hidden layer based on the correlation coefficient (*r*) value. This software has the option for which the maximum layer is limited. Nine different types of networks (i.e., 0, 2, 4, 6, 8, 10, 12, 15, and 25 hidden layers) were constructed by the training set to optimize the number of hidden layers of a network. The test set was converted using each network. We calculated the root–mean–square error (*RMSE*) for nine types of network as follows using Eq. (2):

2 $$RMSE = \sqrt {\frac{{\mathop \sum \nolimits_{i = 1}^{N} \left( {Y_{i} - G_{i} } \right)^{2} }}{N}}$$

where *N* is the number of training cases; *Y*_*i*_ is the converted value (output value) of the UAV-survey data in mesh *i* ($\upmu$Sv h^−1^: air dose rate at 1 m agl.); and *G*_*i*_ is the ground-based survey value (V1) in mesh *i* ($\upmu$Sv h^−1^).

Second, we verified how many data cases can construct a network to converge the error between the ANN conversion value and the ground-based survey value using a limited training set. An ANN was constructed by randomly selecting data from a limited training set hereinafter referred to as “random training.” Two different kinds of data set were selected to evaluate the required number of training cases. The random training network was constructed by a limited training set (i.e., TD1 and TD2) (Table 2). The distribution of each random training set shown in Fig. 4 was selected by considering the radiation intensity or flight altitude of the test set. A random training dataset was extracted from the training (Fig. 4a) and test (Fig. 4b) sets. The number of training cases used for learning ranged from 50 to 1000 (50, 100, 200, 300, 400, 500, 750, and 1000). A random training network was constructed with the same settings. A total of 500 cases of data, other than those selected as the training set, were converted by the random training network to calculate the *RMSE* between the converted and ground-based survey values.

**Table 2:** Limited training set for evaluating the neural network condition.

Data type	Total count rate* (cps)	P5 (m)	T1 ($\upmu$Sv h^−1^)	Number of cases
All training cases	370–19,099	43–244	0.064–13	41,378
TD1	0–5000	50–60	0.12–3.5	2469
TD2	5000–10,000	50–60	0.39–6.8	1544

*Sum of P1, P2, P3, and P4.

### ML-EM method

The detailed procedure of the ML-EM method is given in the paper of Sasaki et al. (2019)^10,11^. The ML-EM method is a type of inverse estimation method that employs radiation information from various directions. The ML-EM method is theoretically expressed as follows by Eq. (3):

3 $$\lambda_{j}^{{\left( {k + { }1} \right)}} = { }\frac{{\lambda_{j}^{\left( k \right)} }}{{\mathop \sum \nolimits_{{i = { }1}}^{D} C_{ij} }}\mathop \sum \limits_{{i = { }1}}^{D} \frac{{y_{i} C_{ij} }}{{\mathop \sum \nolimits_{{j = { }1}}^{B} C_{ij} \lambda_{j}^{\left( k \right)} }}$$

where, *k* is the number of calculation iterations; *j* is the calculation position on the ground; *B* is the total number of ground-based calculation positions; *i* is the detection position; and *D* is the total number of detection positions. $\lambda$ is the calculated intensity value on the ground. *y*_*i*_ is the measured count rate which is corrected by the detector response of radiocesium point source. *C*_*ij*_ is the parameter containing the attenuation coefficient. *C*_*ij*_ accounts for the attenuation by air, soil, and forest and is expressed as follows:

4 $$C_{ij} = F_{x} \left( x \right) \cdot F_{\theta } \left( \theta \right) \cdot F_{h} \left( h \right)$$

5 $$F_{x} \left( x \right) = x^{{ - \alpha_{1} }} \left( {x \le 200} \right)$$

6 $$F_{x} \left( x \right) = \frac{{200^{{ - \alpha_{1} }} \exp \left( { - \alpha_{2} x} \right)}}{{\exp \left( { - 200 \alpha_{2} } \right)}} \left( {200 < x} \right)$$

7 $$F_{\theta } \left( \theta \right) = 1 + A {\text{exp}}\left[ { - \left( {\left( {\theta - \gamma } \right)/\sigma } \right)^{2} } \right]$$

8 $$F_{h} \left( h \right) = \exp \left( { - \varphi h} \right)$$

*F*_*x*_ is the attenuation rate due to air *F*_*x*_ and is calculated from the distance *x* (m) between *i* and *j* by using the particle and heavy ion transport code system (PHITS) developed by the Japan Atomic Energy Agency^20^. *F*_*x*_ was obtained from the decrease in the number of incident photons according to the distance from the point source of ^137^Cs. The calculation result yielded 1.97 and 0.010 as parameters $\alpha_{1} \;{\text{and }}\alpha_{2}$, respectively. *F*_*θ*_ is the attenuation rate due to the effect of the soil scattering calculated from the angle *θ* (deg) formed by *i*–*j* and the soil surface. Parameters *A*, $\gamma \;{\text{and}}\;\sigma$ corresponded to the values of − 0.727, − 35.2, and 34.5, respectively. *F*_*θ*_ was evaluated using the PHITS from the decrease in the number of incident photons when the angle between the soil surface and *i–j* was changed upon centering on the point source of ^137^Cs. *F*_*h*_ is the attenuation rate due to forest shielding calculated from the distance *h* (m) that passes through the forest area between *i* and *j*. *h* is calculated using the DEM and the DSM. Accordingly, 0.061 was used for parameter $\varphi$. Our previous research on the relationship between the air dose rate by the ground-based survey and the UAV-survey in the forest area^11^ showed that *F*_*h*_ depended on the tree height.

### FSM method

The count rate obtained by a dedicated UAV-survey detector (*C*_*all*_: 50–2800 keV) was converted into the air dose rate (*Y*) using a conversion factor (*CD*: 0.00028 $\upmu$Sv h^−1^ cps^−1^) and an attenuation factor (*AF*: 0.0061 m^−1^). The *CD* was obtained in advance by comparing the count rate in the calibration area at altitude *H*_*std*_ (m) with that measured using a survey meter on the ground. The *AF* was calculated from the data obtained by varying the flight altitude on the ground from 20 to 150 m. Equation (9) shows the conversion expression in mesh *i*.

9 $$Y_{i} = \left( {C_{all i} - C_{BG} } \right) \cdot CD \cdot exp\left[ { - AF\left( {H_{std i} - H_{m i} } \right)} \right]$$

where *C*_*BG*_ represents the background counts, including the cosmic ray and the natural radionuclides in the detector crystal, and *H*_*m*_ (m) is the flight altitude above the ground.

### Mapping

Mapping was performed by supplementing unmeasured areas via the interpolation of the measured results. Various methods (e.g., kriging and spline approaches) were proposed for the interpolation, but the kriging method, which linearly assigns weights to the values of the measurement points and in inverse proportion to the distance, was applied herein to the UAV-survey data. The kriging method is easy to use when analyzing a large amount of data because its parameter setting is simple^7^. The interpolation processing was conducted using ArcGIS software (Environmental Systems Research Institute Inc., California, USA). The spatial resolution of the resulting contour map for the air dose rate was 10 m × 10 m.

### Validation method

The ground-based survey data (*G*) were compared with the air dose rate calculated using the UAV-survey (*Y*). Both data were compared by visualizing the unevenness using a scatter diagram. The relative deviation (*RD*) of each measurement cell was calculated as follows to evaluate the accuracy of the scheme used in this study:

10 $$RD_{i} = \left( {Y_{i} - G_{i} } \right)/G_{i}$$

The calculated *RD*s were used to evaluate the total error and the statistical uncertainty shown as a histogram of frequency.

## Results and discussion

### Network construction

**Figure 5:**
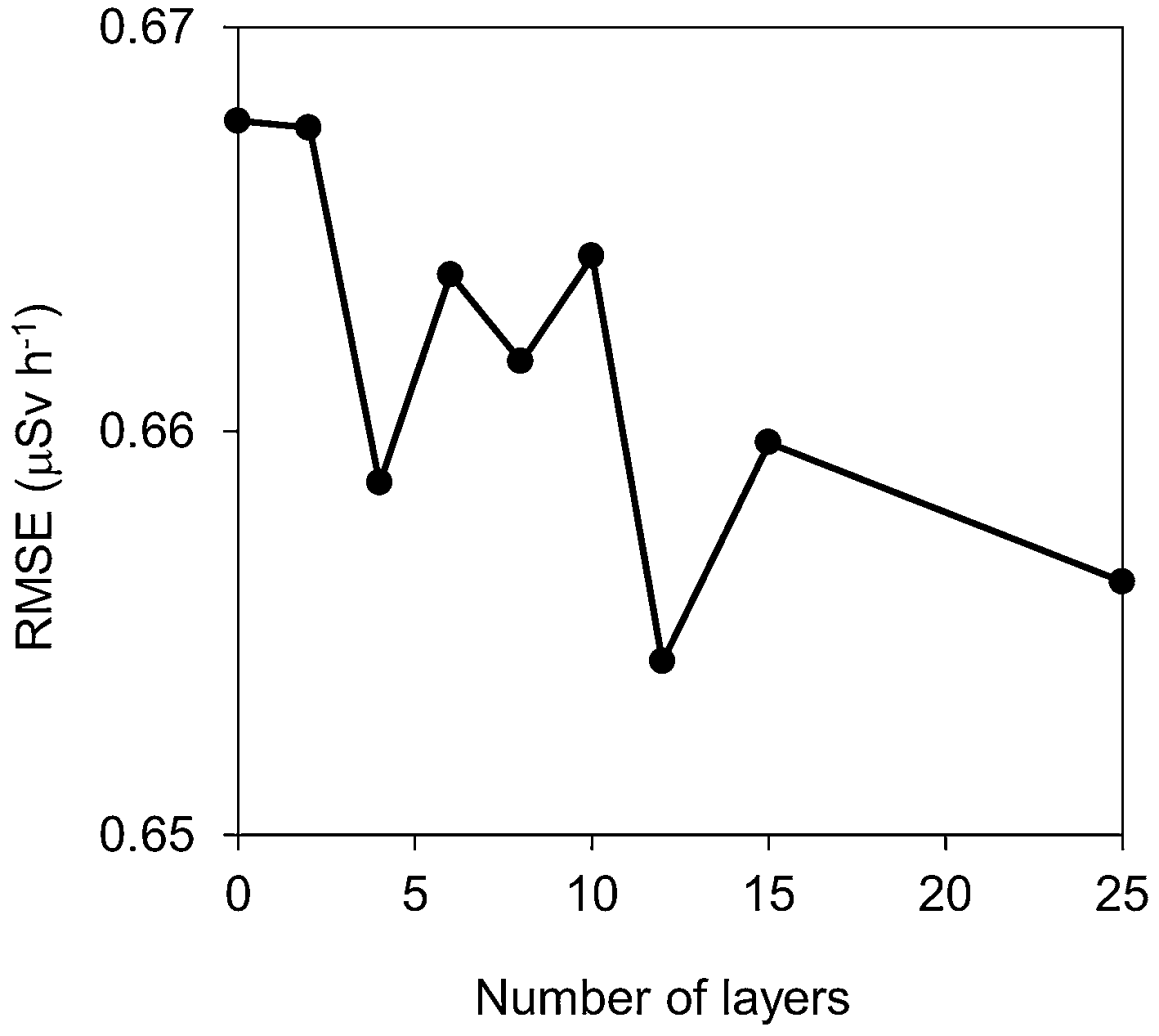
*RMSE* of the ground-based survey value (V1) and the ANN conversion values when the number of hidden layers increases. This is the result of the test set converted to the ANN constructed by the training set.

Figure 5 shows the *RMSE* of the ground-based survey value (T1) and the output values under the condition of hidden layers in the network. The *RMSE* value tended to decrease with the layer increase, although some variation exists. This result suggests that the conversion accuracy improved as the number of hidden layers increased. In the case herein, a network with 12 hidden layers was most suitable for conversion from the UAV-survey data to the air dose rate on the ground. From this result, the network shown in Fig. 2 was constructed with 12 hidden layers for the test set calculation.

### Number of training cases

**Figure 6:**
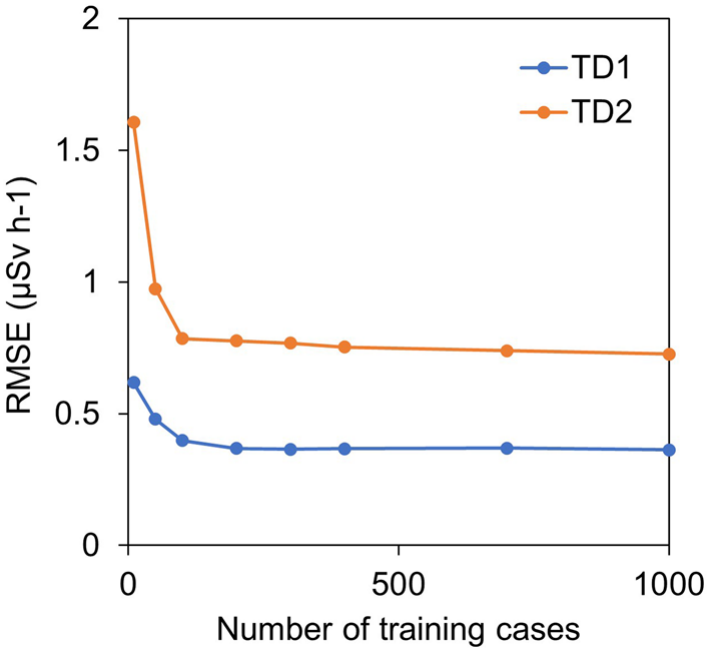
Relationship between the number of training cases and the error. This is the *RMSE* of the ground-based survey value and the ANN conversion values when the number of training cases increases in a random training network. TD1 and TD2 were constructed with the dataset shown in Table 2.

Two types (i.e., TD1 and TD2) of the random training set were created to evaluate the relationship between the number of training cases and the error between the output value by the ANN and the ground-based survey value. The *RMSE* was calculated by comparing the air dose rate with the ground-based survey data that differed from the random training set as an evaluation index. Figure 6 shows the *RMSE* variation with an increasing number of random training cases. Both *RMSE*s converged when the number of training cases was approximately 200 or more in random training. The *RMSE* with two types of different training data sets saturated at 200; thus, this number of training case was considered as a rough criterion for the required number of training data set. In addition, the RMSE values were saturated with a different value, which was expected to affect the statistics error depending on the radiation intensity.

### Validation of the ANN method

**Figure 7:**
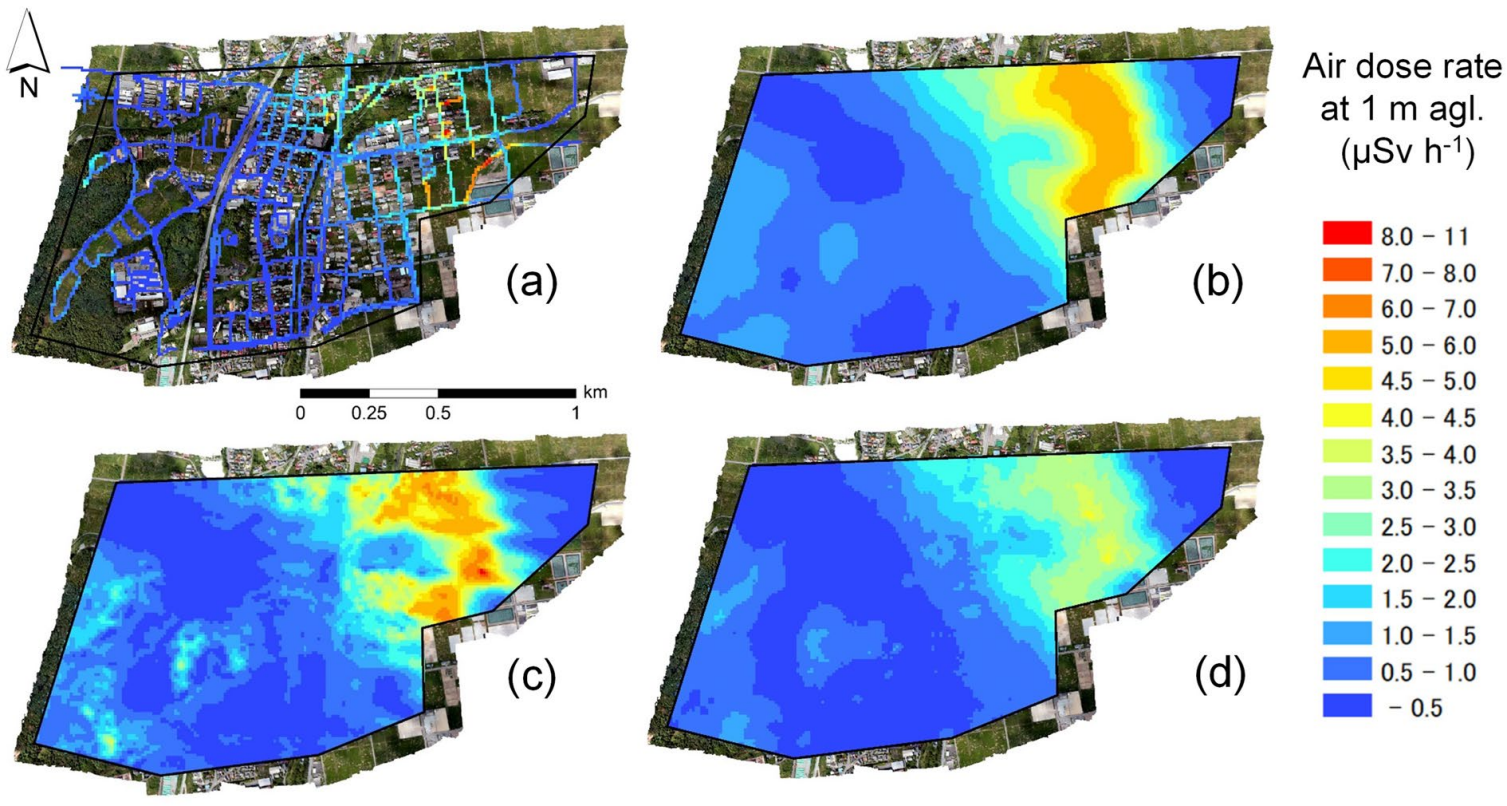
Air dose rate maps at 1 m agl. by (**a**) ground-based survey, (**b**) UAV-survey using the FSM, (**c**) UAV-survey using the ML-EM, and (**d**) UAV-survey using the ANN. This map was created using ArcGIS 10.5. https://www.esri.com/ja-jp/arcgis/about-arcgis/overview.

Figure 7a shows a 10 m mesh contour map of the air dose rate at 1 m agl. of the ground-based survey (V1). Figure 7b–d illustrate the results of converting the measurement values in sky to air dose rates at 1 m agl. using the FSM and those when using the ML-EM and the output value of using the ANN, respectively. This study area is a known path of the radioactive plume at the time of the accident^6^. The east side used as a residential region of this study area had a relatively high air dose rate of 2.0–10 $\upmu$Sv h^−1^), while the forest region in the western area had a relatively low air dose rate of 0.5–3.0 $\upmu$Sv h^−1^. The incline of the air dose rate for this study area was large compared with those from the other regions surrounding the FDNPP.

**Figure 8:**
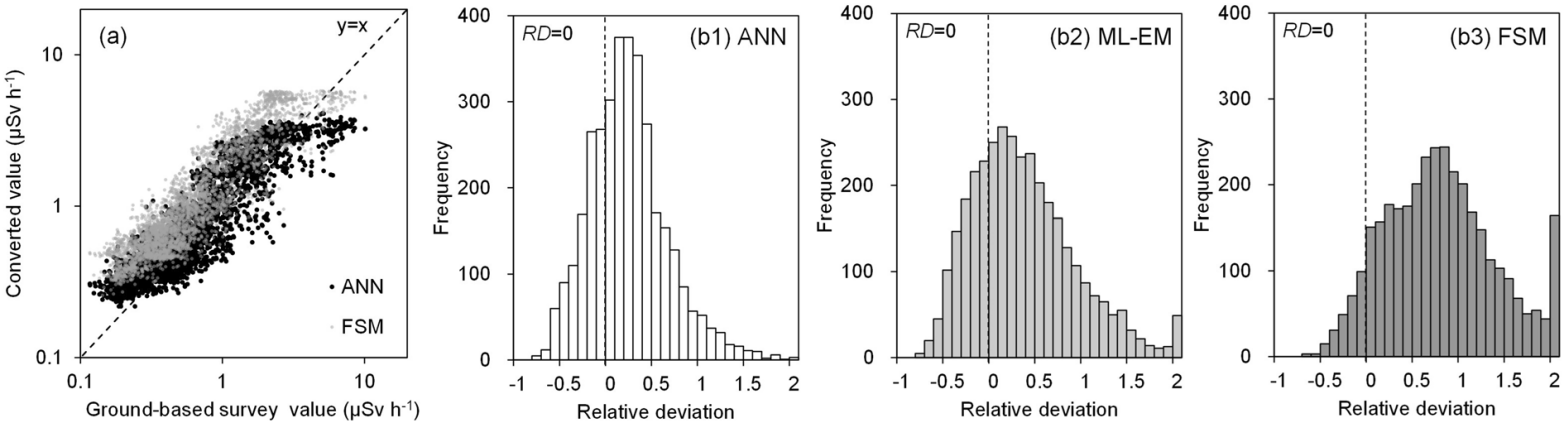
Comparison of the ground-based survey values and the three types of converted values (i.e., ANN, ML-EM, and FSM): (**a**) scatter diagrams of the ground-based survey value and the ANN and FSM values in the same location; and (**b**) histograms of the RDs (i.e., ANN, ML-EM, and FSM).

Figure 8 shows a comparison of the ground-based survey values (V1: Fig. 7a) and the three types of converted values (i.e., ANN, ML-EM, and FSM). The panel is a scatter diagram of the ANN and FSM values and the ground-based survey values in the same location. Three types of UAV-survey conversion values were correlated with the ground-based survey. The FSM tended to be relatively higher than the ground-based survey. The test set area in Fig. 7 was decontaminated in some areas after the accident; hence, the air dose rate at the decontamination area was locally decreased compared with the surroundings. In this case, the UAV-survey accuracy was expected to be affected by the changing field of view of the radiation detector^21^. On the contrary, the ML-EM method used the surrounding topographical and multiple measurement information. Therefore, the bias expressed as the *RMSE* or *RD* was better than the FSM. The ANN bias was reduced in the learning process to improve the error between the ground-based survey and output values. Figures 8(b1–3) present the *RD* histograms for quantitatively evaluating this tendency. Table 3 shows the RMSE with the ground-based survey and the interquartile ranges of the RD (i.e., 25%, 50% and 75%). The 50% interquartile range of the ANN (0.22) was near zero compared with the other two. In addition, the ANN had the smallest RMSE value, indicating that it was best in reproducing the ground-based survey value.

**Table 3:** Evaluation result of the converted value of the UAV-survey data in the FSM, ML-EM, and ANN. All data numbers are 3442.

Parameters		ANN	ML-EM	FSM
Metrics	Range (%)			
RMSE		0.66	0.85	1.00
Interquartile range of *RD*	25	− 0.04	− 0.04	0.37
	50	0.22	0.30	0.78
	75	0.47	0.70	1.18

**Figure 9:**
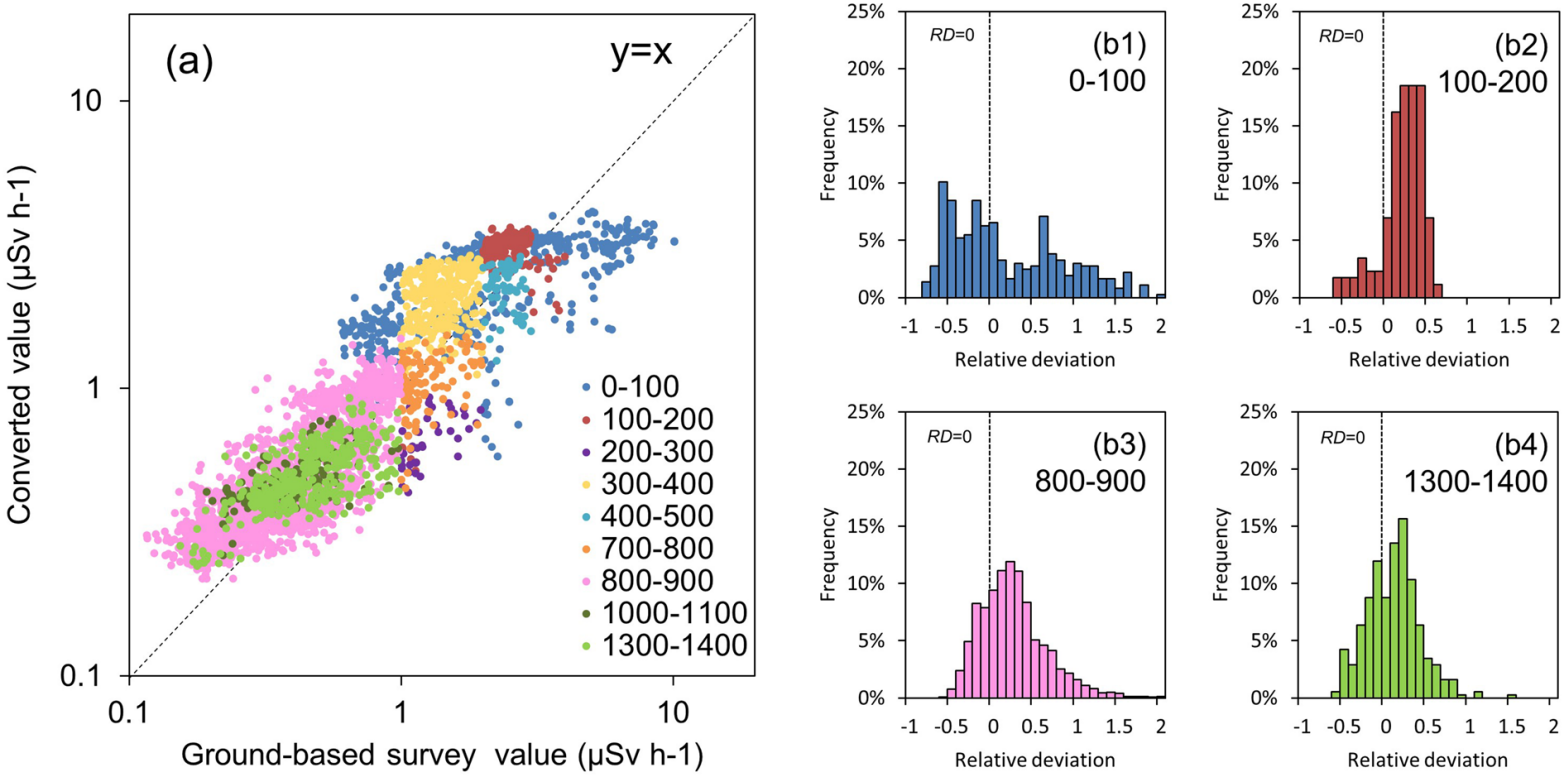
Comparison of the ground-based survey values and the ANN conversion values for the number of training cases in the ANN of Fig. 7d: (**a**) scatter diagrams of the ground-based survey value and the ANN value; and (**b**) histograms of the *RD* for each number of training cases.

For a detailed evaluation of the ANN accuracy, the correlation with the ANN result and the number of training cases were evaluated and shown in Fig. 9. Figures 9(b1–4) depict examples for the correlation with the number of training cases and the *RD* of the ANN and ground-based values. The number of training cases was classified and counted every 10 m at altitude, every 5000 cps of the total count (P1 + P2 + P3 + P4), and every 1 μSv h^−1^ of the ground-based survey value (T1). The histograms show a few changes in the *RD* and *RMSE* with the change of the number of training cases. Table 4 presents the *RMSE* between the ground-based survey value and the ANN value, and the interquartile ranges of 25%, 50%, and 75% to quantitatively evaluate this tendency. The *RMSE* decreased as the number of training cases increased. As a result, the training case number which is necessary to calculate reliable converted value was 200 as well as the simulated verification as shown in Fig. 6. The tendency of the accuracy with the number of training cases was clarified from this application to the field data.

**Table 4:** Evaluation result of the ANN value for each number of training cases. All data numbers are 3442.

Number of training cases		0–100	100–200	200–300	300–400	400–500	700–800	800–900	1000–1100	1300–1400	All data
RMSE		1.72	0.78	0.65	0.77	0.52	0.35	0.18	0.13	0.15	0.66
Inter-quartile range	25%	− 0.36	0.12	− 0.53	0.28	− 0.18	− 0.30	0.01	0.08	− 0.09	− 0.04
	50%	0.02	0.28	− 0.48	0.44	− 0.01	− 0.15	0.24	0.22	0.16	0.22
	75%	0.70	0.41	− 0.41	0.69	0.07	− 0.03	0.49	0.43	0.32	0.47
Data number		366	173	39	302	60	109	1849	167	377	3442

## Conclusion

In this study, we attempted to establish a new conversion method of the UAV-survey using published ANN software. The big data of the UAV-survey that we acquired after the FDNPP accident was used as the ANN training set. Constructing an ANN using the training set around the FDNPP resulted in an optimized hidden layer with 12 layers. The number of training cases was related to the error between the ground value and the ANN-converted value. The evaluation index between the ANN-converted value and the ground-based survey converged at 200 training cases; hence, this number was considered as a rough criterion of the required minimum number. It is thought that the number of training cases can be used as an index to evaluate the reliability of the ANN-converted value. The dose rate map made by the ANN method reproduced ground-based survey results much better than the traditional methods. However, this study used only the basic input variables in the radiation measurement and did not use terrain and photographic data as the input variables. The conversion accuracy is expected to be improved when these input variables are added. Future research will focus on the application of ANN conversion using terrain and photographic data and evaluate the effect of each input variable on conversion.

## Data Availability

The DEM data set we used in the study can be found on the web site of Geospatial Information Authority of Japan^17^. The UAV-survey data set we used in the study can be found on web site of Nuclear Regulation Authority^22^. All data and the code that support the results within this paper and other findings of this study are available from the corresponding author upon reasonable request.
